# Systemic inflammation response index: A good predictor of 30-day all-cause mortality in patients with acute exacerbation of chronic obstructive pulmonary disease

**DOI:** 10.1097/MD.0000000000050912

**Published:** 2026-10-02

**Authors:** Yahu Bai

**Affiliations:** a Department of Critical Care Medicine, Shandong Provincial Third Hospital, Cheeloo College of Medicine, Shandong University, Jinan, China.

**Keywords:** acute exacerbation of chronic obstructive pulmonary disease, MIMIC-Ⅲ database, prognosis, systemic inflammation response index

## Abstract

This study aims to explore the association between the systemic inflammation response index (SIRI) and the prognosis of patients with acute exacerbation of chronic obstructive pulmonary disease (AECOPD) by retrospectively analyzing clinical data derived from the Medical Information Mart for Intensive Care-III (MIMIC-III) database. A total of 426 AECOPD patients were enrolled in this study, among whom 113 died during the 30-day follow-up period, resulting in a mortality rate of 26.6%. Compared with the survival group, patients in the deceased group had higher levels of SIRI (4.19 [2.19-7.68) versus 6.25 (3.09–15.57) ×10^9^/L, *P*<.001]. X-tile software was utilized to determine the optimal SIRI cutoff value of 10.56 × 10^-9^/L for predicting 30-day all-cause mortality. Patients were stratified into 2 groups based on this cutoff value: a low SIRI group (<10.56 × 10^9^/L) and a high SIRI group (≥10.56 × 10^9^/L). Baseline characteristic comparisons demonstrated that patients in the high-SIRI group presented significantly higher 30-day, 90-day, 1-year, and in-hospital mortality than those in the low-SIRI group (all *P* < .05). After adjustment for potential confounders, the Cox proportional-hazards regression model identified elevated SIRI as an independent risk factor for 30-day all-cause mortality (HR = 1.743, 95%CI: 1.169-2.598, *P* = .006), and subgroup analyses confirmed the robustness of this correlation. Additionally, ROC curves showed that the area under the curve (AUC) for SIRI was greater than that for white blood cells and neutrophils (AUC 0.612 vs 0.584; 0.612 vs 0.557), but slightly smaller than that for sequential organ failure assessment (AUC 0.612 vs 0.619). Collectively, our findings indicate that SIRI represents a promising novel inflammatory biomarker for predicting 30-day all-cause mortality among patients with AECOPD.

## 1. Introduction

Chronic obstructive pulmonary disease (COPD) is a common disease in the field of respiratory medicine and is characterized by high morbidity and mortality rates, which pose a significant burden to the economy and society. According to the Burden of Obstructive Lung Disease (BOLD) study and other large-scale epidemiological investigations, the global prevalence of COPD reaches 10.3% (95% CI: 8.2%–12.8%).^[1,2]^ Meanwhile, the World Health Organization (WHO) explicitly stated that COPD had become the third leading cause of death worldwide in 2021.^[3]^With increasing smoking prevalence in developing countries and population aging across high-income nations, the health threat posed by COPD will continue to escalate. Reports indicate that from 1990 to 2019, over the span of 30 years, both the number of cases and deaths due to COPD worldwide have significantly increased.^[4]^ Additionally, it is projected that the prevalence of COPD will further rise over the next 40 years, with the annual number of deaths due to COPD and its related diseases potentially exceeding 5.4 million by 2060.^[5]^ Therefore, the prevention, treatment, and management of COPD patients should be a high priority for every healthcare professional.

The 2023 revision of the Global Initiative for Chronic Obstructive Lung Disease (GOLD) has updated the definition of acute exacerbation of chronic obstructive pulmonary disease (AECOPD). AECOPD is characterized by events occurring within 14 days that feature increased shortness of breath and/or cough with sputum production, which may be accompanied by dyspnea and/or tachycardia. These events are typically associated with increased local and systemic inflammation due to infections, pollution, or other airway-damaging factors.^[6]^ Acute exacerbation of COPD is a common clinical event in the natural course of COPD,^[7]^ with reports indicating that patients with COPD experience acute exacerbations an average of 0.5 to 3.5 times per year.^[8]^ Since AECOPD aggravates overall disease severity and contributes to irreversible long-term decline in pulmonary function,^[9]^ it not only significantly increases mortality among COPD patients but is also closely related to decreased quality of life, loss of work capacity, and increased medical expenses. One of the main treatment goals for COPD is to prevent and reduce acute exacerbations; however, unfortunately, there are currently no reliable clinical indicators, physiological, radiological, or biological markers that can accurately predict the occurrence of AECOPD.^[10]^ Under this clinical background, exploring prognostic determinants for AECOPD, identifying high-risk patients in a timely manner, and implementing proactive therapeutic interventions may help reduce COPD-related mortality.

It is well known that abnormal inflammatory responses play an important role in the occurrence and development of chronic obstructive pulmonary disease (COPD). Some scholars have suggested that the airflow obstruction in COPD is a result of chronic pulmonary inflammation, which is mediated by various inflammatory cells, including macrophages, neutrophils, T lymphocytes, and B lymphocytes, which fill the airway lumen of COPD patients.^[11]^At the same time, generally speaking, an individual’s systemic inflammatory status is reflected in the changes in the relative levels of peripheral blood leukocytes in circulation, mainly manifested through variations in neutrophils, monocytes, and lymphocytes.^[12]^ These inflammatory cells are widely used in clinical practice due to their significant role in the occurrence and development of inflammation. However, the reference significance of single indicators is relatively limited. Therefore, in recent years, there has been a tendency to combine multiple indicators into a new comprehensive inflammatory index, such as the neutrophil-to-lymphocyte ratio (NLR), lymphocyte-to-monocyte ratio (LMR), and platelet-to-lymphocyte ratio (PLR). The clinical significance of these indicators has been validated in various diseases, including solid tumors, systemic lupus erythematosus, coronary artery disease, retinal artery occlusion, and chronic kidney disease, among others.^[13–19]^

In addition to the aforementioned comprehensive inflammatory indices, in 2016, Qi Qi et al^[20]^ first proposed a composite index based on absolute counts of 3 different inflammatory cells (i.e., neutrophils, monocytes, and lymphocytes), called the systemic inflammation response index (SIRI) (SIRI = neutrophil count × monocyte count/ lymphocyte count). Their original study confirmed that SIRI could influence clinical outcomes of patients with advanced pancreatic cancer and could mirror both local immune responses and systemic inflammatory burden. Since then, SIRI has been found to be highly correlated with the prognosis of various diseases. Hui Dang et al ^[21]^ confirmed that elevated SIRI is associated with mortality in patients with acute ischemic stroke using data from the MIMIC-IV database. Similar conclusions have been supported by other studies as well.^[22]^ Xue Wang et al^[23]^ conducted a statistical analysis involving 3964 patients from the MIMIC-III database and 261 patients from their institution, ultimately concluding that SIRI could be an important factor in predicting all-cause mortality in elderly heart failure patients. Nevertheless, the prognostic significance of SIRI in patients suffering from AECOPD remains largely unexplored. Hence, this present study aimed to evaluate the relationship between SIRI and clinical prognosis in AECOPD patients.

## 2. Materials and methods

### 2.1. Data source

Clinical data for this retrospective observational study were extracted from the Medical Information Mart for Intensive Care III version 1.4 (MIMIC-III) database. The MIMIC-III database was developed collaboratively by the Massachusetts Institute of Technology’s Laboratory for Computational Physiology, Beth Israel Deaconess Medical Center, and Philips Healthcare. It is a free, large, single-center dataset of intensive care unit (ICU) medical data, containing 53,423 hospital admissions of adult patients (aged ≥ 16) from 2001 to 2012.^[24]^ The dataset includes demographic characteristics, vital signs, laboratory test results, nursing records, medication details, diagnosis records, surgical records, length of stay, and survival data. The author has completed the Protecting Human Research Participants training program organized by the National Institutes of Health and obtained the Collaborative Institutional Training Initiative (CITI) certificate (ID: 48237440).

### 2.2. Patient selection

The patients included in this study were all recruited from the MIMIC-III v1.4 database. The inclusion criteria were: patients diagnosed with an AECOPD with the diagnosis code 49,121 according to the ninth revision of the International Classification of Diseases (ICD-9); and patients who were admitted to the ICU for the first time. The exclusion criteria were: patients younger than 18 years old; patients with an ICU length of stay of <24 hours; patients with missing SIRI and key information; and patients with hematological diseases or tumors.

### 2.3. Data extraction

The data were extracted from the MIMIC-III database using the database management software pgAdmin 4 and Structured Query Language (SQL). The extracted data included: demographic characteristics: age, gender, race, admission time, discharge time, ICU admission time, ICU discharge time, survival status, and time of death; laboratory test results (the first test results after ICU admission): white blood cell count, percentage of neutrophils, percentage of monocytes, percentage of lymphocytes, platelet count, hemoglobin, serum glucose, serum creatinine, serum urea nitrogen, pH (Pondus hydrogenii), arterial oxygen tension (PaO_2_), and arterial carbon dioxide tension (PaCO_2_); comorbidities: congestive heart failure, valvular heart disease, peripheral vascular disease, hypertension, complicated diabetes, renal failure, liver disease, and rheumatoid arthritis; vital signs (average values within 24 hours of ICU admission): heart rate, systolic blood pressure, diastolic blood pressure, mean arterial pressure, respiratory rate, and temperature; and scoring systems: sequential organ failure assessment (SOFA) and Simplified Acute Physiology Score II (SAPS II).

### 2.4. Clinical outcomes

Clinical outcomes include the length of ICU stay (LOS ICU), length of hospital stay (LOS hospital), 30-day all-cause mortality, 90-day all-cause mortality, 1-year all-cause mortality, and in-hospital mortality. The primary outcome was 30-day all-cause mortality. The secondary outcomes were 90-day mortality, 1-year all-cause mortality, and in-hospital mortality of patients with AECOPD.

### 2.5. Management of missing data

No missing values existed for either primary or secondary outcomes. To minimize bias introduced by missing data, variables with missing proportions exceeding 15% were excluded from statistical analyses. Median imputation was applied for continuous variables with partial missing entries.

### 2.6. Statistical analysis

For continuous variables, normality was first assessed using the Kolmogorov-Smirnov test. If the data followed a normal distribution, it was expressed as the mean with standard deviation, and intergroup comparisons were made using independent samples *t* tests. If the data did not follow a normal distribution, it was expressed as the median with 25 to 75th percentile, and intergroup comparisons were made using the Mann–Whitney *U* test. For categorical variables, results were expressed as counts (percentages), and intergroup comparisons were conducted using the chi-square test.Data organization and statistical analysis were conducted using STATA 17 and SPSS 26, with all tests being two-tailed and a significance level set at *P* < .05.

First, we compared baseline characteristics between the 30-day survivor group and non-survivor group of included patients. X-tile software (Yale School of Medicine, version 3.6.1) was then employed to identify the optimal SIRI cutoff point for predicting 30-day all-cause mortality, and study subjects were further divided into low-SIRI and high-SIRI groups for subsequent analyses. Univariate and multivariate Cox proportional-hazards regression models were constructed to evaluate the associations between SIRI and 30-day, 90-day, 1-year all-cause mortality as well as in-hospital mortality in AECOPD patients. Results were presented as hazard ratios (HR) with 95% confidence intervals (CI). Three models were constructed: Model 1 was unadjusted for any covariates, Model 2 adjusted for age, gender, and race, and Model 3 adjusted for age, gender, race, hemoglobin, blood urea nitrogen, valvular heart disease, hypertension, rheumatoid arthritis, heart rate, systolic blood pressure, and diastolic blood pressure. To verify the stability of research findings, SIRI values were further categorized into quartiles to explore the correlations between different SIRI quartile strata and mortality outcomes. Survival analysis was conducted using the Kaplan–Meier method to plot survival curves, with comparisons made using the log-rank test.

Stratification was performed based on age (<73 years and ≥ 73 years), sex, race, hemoglobin (<10.9 g/dL and ≥ 10.9 g/dL), blood urea nitrogen (<25 mg/dL and ≥ 25 mg/dL), valvular heart disease, hypertension, and rheumatoid arthritis. Cox proportional-hazards regression models were fitted within each subgroup to evaluate the prognostic effect of SIRI and detect potential interaction effects. Receiver-operating characteristic (ROC) curves were plotted to evaluate the predictive performance of SIRI for 30-day all-cause mortality in AECOPD patients; the area under the curve (AUC) was calculated to compare the discriminative ability of SIRI against leukocyte count, neutrophil count, and SOFA score.

## 3. Results

### 3.1. Clinical characteristics of AECOPD patients in the survival group and death group within 30 days

In total, 426 AECOPD patients satisfying the predefined eligibility criteria were extracted from the MIMIC-III database. Figure 1 illustrates the flowchart for study cohort screening. After 30 days of follow-up, a total of 113 patients died, and the differences between the survival group and the death group are shown in Table 1. Compared to the survival group, the death group had a higher age, higher white blood cell counts, lower lymphocyte counts, lower hemoglobin levels, and higher blood urea nitrogen levels. Additionally, the death group had lower systolic blood pressure, higher SOFA scores, higher simplified acute physiology score II scores, and higher SIRI values. In addition to these indicators, there were also statistically significant differences in race and comorbid hypertension (*P* < .05). No obvious differences were detected for the remaining variables between the 2 groups.

**Table 1:** Characteristics of AECOPD patients according to 30-day all-cause mortality.

Characteristic	Survivors(n = 313)	Non-survivors(n = 113)	*P*-value
**Demographics**			
Age, years	71.10 (63.40–79.49)	76.839 (69.97–82.48)	<.001
Gender, n(%)			.678
Male	148 (47.3)	56 (49.6)	
Female	165 (52.7)	57 (50.4)	
Ethnicity, n(%)			.024
White	237 (75.7)	79 (69.9)	
Black	28 (8.9)	5 (4.4)	
Other	48 (15.3)	29 (25.7)	
**Comorbidities,** n(%)			
Congestive heart failure	171 (54.8)	67 (59.3)	.393
Valvular disease	38 (12.1)	20 (17.7)	.140
Peripheral vascular disease	32 (10.2)	12 (10.6)	.906
Hypertension	186 (59.4)	55 (48.7)	.048
Complicated diabetes	18 (5.8)	5 (4.4)	.593
Renal failure	60 (19.2)	21 (18.6)	.892
Liver disease	22 (7.0)	9 (8.0)	.743
Rheumatoid arthritis	14 (4.5)	3 (2.7)	.397
**Laboratory parameters**			
White blood cell count, 10^9^/L	10.8 (7.9–15.1)	13.0 (8.15–18.9)	.008
Neutrophil count, 10^9^/L	87.5 (80.1–91.9)	89.0 (82.9–93.1)	.074
Monocyte count, 10^9^/L	3.3 (2.0–5.0)	3.3(2.0-5.0)	.808
Lymphocyte count, 10^9^/L	7.0 (4.2–11.3)	5.5 (3.0–9.5)	.001
Hemoglobin, g/dL	11.2 (9.9–12.8)	10.3 (9.1–11.7)	<.001
Platelet count, 10^9^/L	246.0 (183.0–312.0)	233.5 (159.0–316.0)	.169
Serum creatinine, mg/dL	1.0 (0.7–1.5)	1.15 (0.7–1.5)	.301
Blood urea nitrogen, mg/dL	24.0 (17.0–34.0)	31.5 (19.0–50.0)	.001
Glucose, mg/dL	143.0 (118.0–180.0)	143.0 (118.3–183.0)	.802
pH	7.34 (7.27–7.39)	7.34(7.27-7.38)	.900
PaO_2_, mm Hg	98.0 (76.0–140.0)	98.0 (69.3–147.0)	.467
PaCO_2_, mm Hg	53.0 (45.0–69.0)	53.0 (41.3–65.5)	.097
SIRI, 10^9^/L	4.19 (2.19–7.68)	6.25 (3.09–15.57)	<.001
**Vital signs**			
Heart rate, beats/min	89.08 ± 14.91	92.03 ± 17.39	.086
SBP, mm Hg	118.23 (109.38–129.80)	113.57 (104.97–126.21)	.026
DBP, mm Hg	60.70 ± 9.93	58.95 ± 10.11	.117
MAP, mm Hg	76.80 (70.94–83.81)	74.47 (67.88–83.47)	.134
Respiratory rate, times/min	20.50 ± 3.81	20.99 ± 4.30	.255
Temperature, °C	36.70 (36.34–37.04)	36.63 (36.21–37.05)	.472
**Scores**			
SOFA	4.00 (2.00–6.00)	5.00 (3.00–7.00)	<.001
SAPS Ⅱ	37.00 (30.50–44.00)	46.00 (37.00–56.00)	<.001
**Clinical outcomes**			
LOS ICU, day	4.38 (2.23–8.94)	5.21 (2.66–10.57)	.182
LOS hospital, day	9.87 (6.43–15.75)	8.94 (4.62–15.84)	.248

DBP = diastolic blood pressure, LOS hospital = length of hospital stay, LOS ICU = length of ICU stay, MAP = mean arterial pressure, SAPS II = simplified acute physiology score II, SBP = systolic blood pressure, SIRI = neutrophil count × monocyte count/ lymphocyte count, SIRI = systemic inflammation response index, SOFA = sequential organ failure assessment.

**Figure 1.**
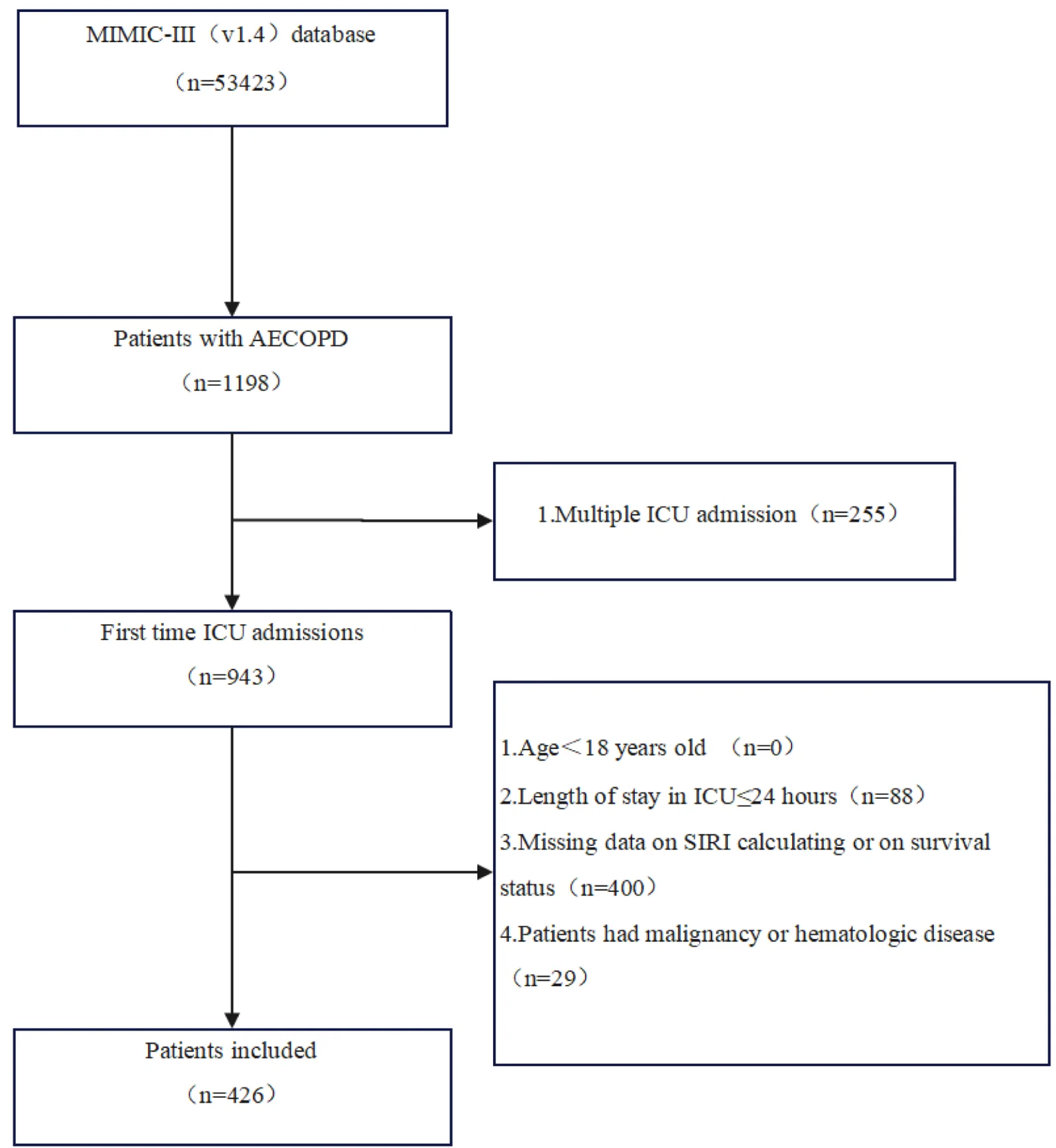
Flow chart of study population selection. AECOPD = acute exacerbation of chronic obstructive pulmonary disease, ICU = intensive care unit.

### 3.2. Clinical characteristics of AECOPD patients with different SIRI levels

All enrolled subjects were dichotomized by the X-tile-derived SIRI cutoff value: 327 patients were assigned to the low-SIRI group (<10.56 × 10^9^/L), and 99 patients were assigned to the high-SIRI group (≥10.56 × 10^9^/L). Baseline differences between the 2 subgroups are summarized in Table 2. Compared with the low SIRI group, patients in the high SIRI group were older and had higher SOFA and simplified acute physiology score II scores. Additionally, there were statistically significant differences in white blood cell count, neutrophil count, monocyte count, lymphocyte count, and platelet count between the 2 groups (*P* < .05). In terms of clinical outcomes, the high-SIRI group exhibited significantly higher 30-day, 90-day, 1-year, and in-hospital mortality rates. In contrast, ICU and hospital length-of-stay showed no statistical difference between groups. No other variables displayed statistically significant disparities.

**Table 2:** Baseline characteristics stratified by SIRI.

Characteristic	SIRI < 10.56 × 10^9^/L	SIRI ≥ 10.56 × 10^9^/L	*P*-value
N (sample size)	327	99	
**Demographics**			
Age, yr	71.66 (63.73–79.70)	75.54 (67.31–82.84)	.008
Gender, n(%)			.892
Male	156 (47.7)	48 (48.5)	
Female	171 (52.3)	51 (51.5)	
Ethnicity, n(%)			.171
White	247 (75.5)	69 (69.7)	
Black	27 (8.3)	6 (6.1)	
Other	53 (16.2)	24 (24.2)	
**Comorbidities,** n(%)			
Congestive heart failure	182 (55.7)	56 (56.6)	.873
Valvular disease	43 (13.1)	15 (15.2)	.611
Peripheral vascular disease	31 (9.5)	13 (13.1)	.296
Hypertension	187 (57.2)	54 (54.5)	.642
Complicated diabetes	17 (5.2)	6 (6.1)	.740
Renal failure	62 (19.0)	19 (19.2)	.959
Liver disease	25 (7.6)	6 (6.1)	.595
Rheumatoid arthritis	13 (4.0)	4 (4.0)	.977
**Laboratory parameters**			
White blood cell count, 10^9^/L	9.9 (7.1–14.3)	18.4 (12.8–24.0)	<.001
Neutrophil count, 10^9^/L	87.0 (79.6–91.2)	90.8 (85.0–94.0)	<.001
Monocyte count, 10^9^/L	3.0 (1.9–4.6)	4.3 (2.8–6.0)	<.001
Lymphocyte count, 10^9^/L	8.0 (5.1–12.1)	3.0 (2.0–4.0)	<.001
Hemoglobin, g/dL	10.9 (9.8–12.6)	10.9 (9.7–12.4)	.769
Platelet count, 10^9^/L	233.0 (175.0–301.0)	273.0 (198.0–366.0)	.005
Serum creatinine, mg/dL	1.0 (0.7–1.5)	1.2 (0.8–1.6)	.153
Blood urea nitrogen, mg/dL	25.0 (17.0–38.0)	27.0 (18.0–43.0)	.263
Glucose, mg/dL	141.0 (117.0–176.0)	148.0 (120.0–194.0)	.209
pH	7.34 (7.28–7.39)	7.34 (7.26–7.39)	.330
PaO_2_, mm Hg	98.0 (76.0–144.0)	96.0 (71.0–123.0)	.060
PaCO_2_, mm Hg	53.0 (44.0–69.0)	53.0 (46.0–68.00)	.753
**Vital signs**			
Heart rate, beats/min	89.17 ± 15.48	92.10 ± 16.03	.103
SBP, mm Hg	116.93 (108.97–128.97)	91.29 (105.88–129.14)	.552
DBP, mm Hg	60.40 ± 9.68	59.64 ± 10.95	.511
MAP, mm Hg	76.50 (70.59–83.89)	75.48 (69.55–82.99)	.445
Respiratory rate, times/min	20.61 ± 3.92	20.65 ± 4.05	.930
Temperature, °C	36.67 (36.32–37.00)	36.71 (36.31–37.17)	.273
**Scores**			
SOFA	4.00 (2.00–6.00)	5.00 (3.00–7.00)	.035
SAPS Ⅱ	38.00 (32.00–46.00)	43.00 (35.00–54.00)	.004
**Clinical outcomes**			
30-day all-cause mortality, n(%)	71 (21.7)	42 (42.4)	<.001
90-day all-cause mortality, n(%)	96 (29.4)	51 (51.5)	<.001
1-year all-cause mortality, n(%)	140 (42.8)	56 (56.6)	.016
in-hospital mortality,n(%)	41 (12.6)	32 (32.3)	<.001
LOS ICU, day	4.35 (2.25–8.93)	4.91 (2.60–10.86)	.307
LOS hospital, day	9.71 (6.07–15.75)	9.36 (6.06–15.62)	.781

DBP = diastolic blood pressure, LOS hospital = length of hospital stay, LOS ICU = length of ICU stay, MAP = mean arterial pressure, SAPS II = simplified acute physiology score II, SBP = systolic blood pressure, SIRI = neutrophil count × monocyte count/ lymphocyte count, SIRI = systemic inflammation response index, SOFA = sequential organ failure assessment.

### 3.3. Cox regression analysis of SIRI and clinical outcomes in AECOPD patients

Univariate and multivariate Cox proportional-hazards regression models were constructed to assess the prognostic association between SIRI and clinical outcomes among AECOPD patients, as shown in Table 3. In Model 1, high SIRI was significantly associated with elevated risks of 30-day, 90-day, 1-year all-cause mortality and in-hospital mortality in AECOPD patients. Such associations remained significant in Model 2 after adjustment for age, gender, and race, and persisted in Model 3 following adjustment for additional confounding variables. After SIRI was stratified into quartiles, Model 1 demonstrated that the fourth quartile, relative to the first quartile, correlated with increased risks of 30-day, 90-day, and in-hospital mortality, whereas the association with 1-year all-cause mortality was nonsignificant. Similar results were obtained in Model 2. After full adjustment for confounders in Model 3, the fourth quartile remained an independent risk factor for 30-day all-cause mortality and in-hospital mortality, while the associations with 90-day and 1-year mortality lost statistical significance.

**Table 3:** Association between SIRI and clinical outcomes in patients with AECOPD.

Clinical outcomes	Model 1		Model 2		Model 3	
	HR(95%CI)	*P*-value	HR(95%CI)	*P*-value	HR(95%CI)	*P*-value
**Primary outcome**						
30-day all-cause mortality						
SIRI < 10.56	1		1		1	
SIRI ≥ 10.56	2.159 (1.473–3.164)	<.001	1.945 (1.320–2.865)	.001	1.743 (1.169–2.598)	.006
SIRI (quartile)						
<2.29 × 10^9^/L	1		1		1	
2.29-4.65	1.197 (0.661–2.166)	.553	1.130 (0.623–2.050)	.658	1.082 (0.589–1.987)	.799
4.65-9.71	1.324 (0.739–2.371)	.346	1.250 (0.695–2.249)	.381	1.176 (0.649–2.133)	.593
≥9.71	2.348 (1.381–3.993)	.002	2.044 (1.193–3.502)	.005	1.771 (1.019–3.077)	.043
*P* for trend		.001		.005		.029
**Secondary outcomes**						
90-day all-cause mortality						
SIRI < 10.56	1		1		1	
SIRI ≥ 10.56	2.049 (1.458–2.880)	<.001	1.886 (1.321–2.635)	<.001	1.706 (1.197–2.431)	.003
SIRI (quartile)						
<2.29	1		1		1	
2.29-4.65	1.005 (0.611–1.653)	.984	0.952 (0.577–1.569)	.847	0.895 (0.538–1.491)	.671
4.65-9.71	1.089 (0.667–1.778)	.733	1.047 (0.639–1.715)	.856	0.941 (0.569–1.555)	.813
≥9.71	1.926 (1.234–3.007)	.004	1.727 (1.099–2.713)	.018	1.472 (0.923–2.346)	.104
*P* for trend		.003		.011		.075
1-year all-cause mortality						
SIRI < 10.56	1		1		1	
SIRI ≥ 10.56	1.572 (1.153–2.145)	.004	1.495 (1.094–2.043)	.012	1.418 (1.028–1.955)	.033
SIRI (quartile)						
<2.29	1		1		1	
2.29-4.65	0.903 (0.597–1.366)	.630	0.847 (0.559–1.283)	.434	0.808 (0.530–1.234)	.325
4.65-9.71	1.008 (0.671–1.515)	.968	0.963 (0.639–1.451)	.856	0.917 (0.604–1.393)	.917
≥9.71	1.410 (0.948–2.069)	.09	1.295 (0.874–1.920)		1.187 (0.790–1.781)	.409
*P* for trend		.068		.134		.288
In-hospital mortality						
SIRI < 10.56	1		1		1	
SIRI ≥ 10.56	2.588 (1.630–4.109)	<.001	2.401 (1.502–3.838)	<.001	2.178 (1.343–3.533)	.002
SIRI (quartile)						
<2.29	1		1		1	
2.29-4.65	1.074 (0.490–2.353)	.859	1.037 (0.472–2.276)	.928	1.083 (0.490–2.394)	.844
4.65-9.71	1.251 (0.585–2.672)	.564	1.238 (0.577–2.658)	.583	1.240 (0.571–2.692)	.587
≥9.71	2.735 (1.412–5.295)	.003	2.506 (1.284–4.892)	.007	2.327 (1.170–4.627)	.016
*P* for trend		.001		.002		.007

Model 1 was adjusted for none.

Model 2 was adjusted for age, gender, and ethnicity.

Model 3 was adjusted for age, gender, ethnicity, hemoglobin, blood urea nitrogen, valvular disease, hypertension, rheumatoid arthritis, heart rate, systolic blood pressure, and diastolic blood pressure.

SIRI = Systemic Inflammation Response Index, HR = hazard ratio, CI = confidence interval.

### 3.4. Kaplan–Meier survival curves of SIRI and clinical outcomes in AECOPD patients

Kaplan–Meier survival curves were generated to compare survival probabilities between high-SIRI versus low-SIRI groups, as well as across SIRI quartiles (Fig. 2). Compared to the low SIRI group, the high SIRI group had significantly lower survival rates at 30 days, 90 days, and 1 year (57.6% vs 78.3%, *P* < .001; 48.5% vs 70.6%, *P* < .001; 43.4% vs 57.2%, *P* < .01). For quartile-stratified analyses, the Kaplan–Meier survival curves showed that the fourth quartile had significantly lower survival rates at 30 days and 90 days compared to the first, second, and third quartiles (*P* < .01), but there were no statistically significant differences in 1-year survival rates among the 4 groups (*P* > .05).

**Figure 2.**
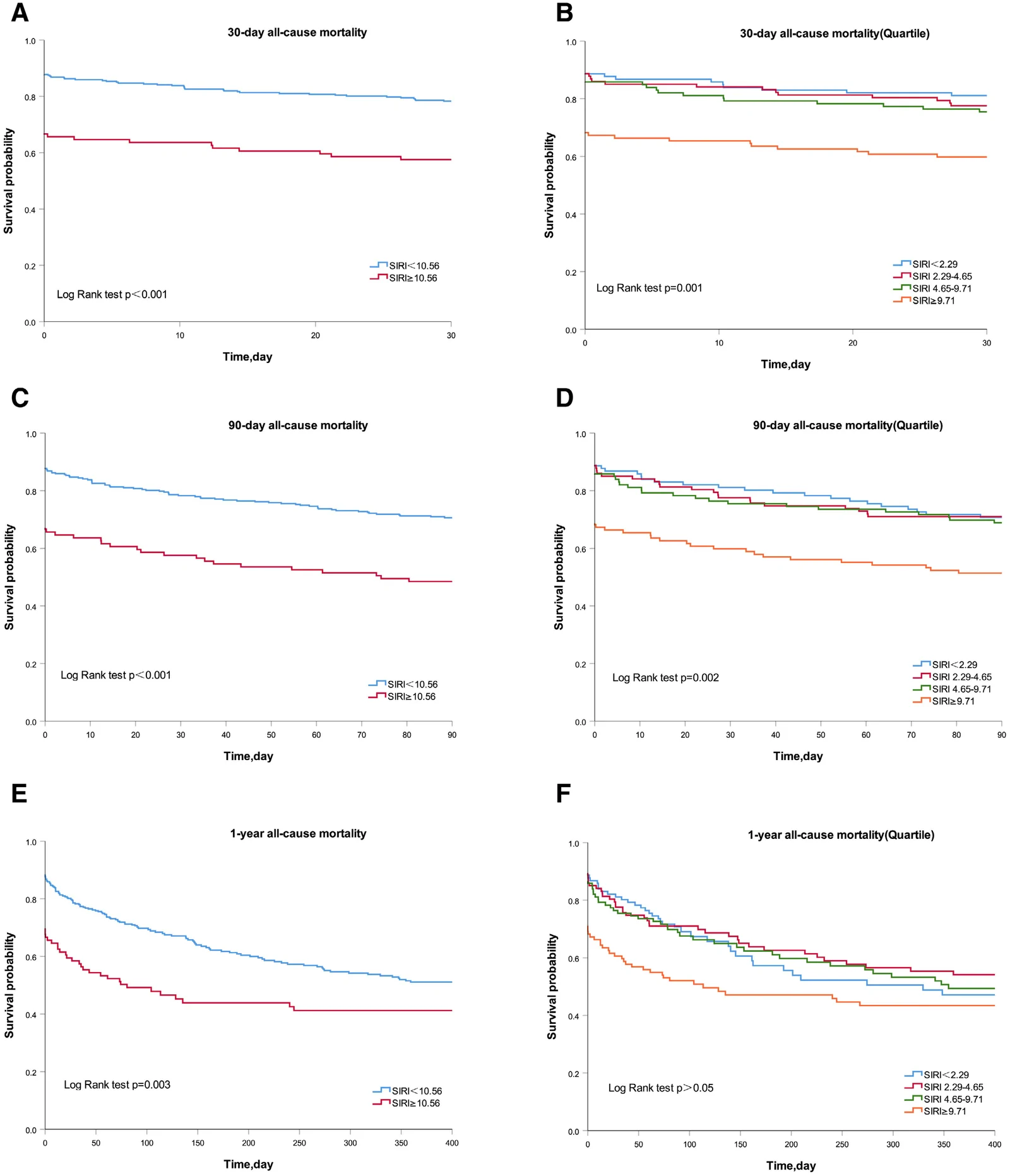
Kaplan–Meier survival analysis plot. SIRI = systemic inflammation response index.

### 3.5. Subgroup analysis of 30-day all-cause mortality in AECOPD patients

Stratified subgroup analyses were performed to verify the stability and consistency of our findings and further evaluate the correlation between SIRI and 30-day all-cause mortality across different clinical subgroups (Table 4). The results indicated that higher levels of SIRI are closely associated with increased 30-day all-cause mortality in AECOPD patients. No statistically significant interaction was detected between SIRI and any stratification factor for 30-day all-cause mortality.

**Table 4:** Subgroup analysis for the effect of SIRI on 30-day all-cause mortality in patients with AECOPD.

	NO.of patients	HR(95%CI)	*P*-value	*p* for interaction
Age, yr				>.99
<73	216	1.977 (0.980–3.988)	.057	
≥73	210	1.972 (1.244–3.127)	.004	
Gender				.678
Male	204	2.353 (1.375–4.029)	.002	
Female	222	1.979 (1.148–3.413)	.014	
Ethnicity				.409
White	316	2.26 (1.429–3.574)	<.001	
Black	33	3.438 (0.571–20.700)	.178	
Others	77	1.546 (0.724–3.259)	.263	
Hemoglobin, g/dL				.231
<10.9	214	1.803 (1.104–2.946)	.019	
≥10.9	212	2.883 (1.554–5.351)	.001	
Blood urea nitrogen, mg/dL				.552
<25	220	1.840 (0.996–3.399)	.052	
≥25	206	2.351 (1.436–3.848)	.001	
Valvular disease				.249
Yes	58	1.284 (0.493–3.342)	.609	
No	368	2.378 (1.565–3.614)	<.001	
Hypertension				.979
Yes	241	2.193 (1.265–3.803)	.005	
No	185	2.089 (1.228–3.557)	.007	
Rheumatoid arthritis				.842
Yes	17	1.625(0.147–17.921)	.692	
No	409	2.182(1.481–3.214)	<.001	

Confounders adjustment were performed as in Model 1. Cox proportional hazards regression models were used to calculate hazard ratio (HR) with 95% confidence interval (CI). The low SIRI level group (<10.56 × 10^9^/L) was set as the reference group.SIRI, systemic inflammation response index; AECOPD, acute exacerbation of chronic obstructive pulmonary disease.

### 3.6. Prognostic value of SIRI and other parameters

ROC curves were plotted to quantify the predictive performance of SIRI. We first compared SIRI with white blood cell count and neutrophil count, and subsequently compared SIRI with the SOFA score (Fig. 3). SIRI exhibited superior predictive power for 30-day all-cause mortality of AECOPD patients compared with white blood cell count and neutrophil count (AUC 0.612 vs 0.584; 0.612 vs 0.557), with slightly inferior performance relative to SOFA (AUC 0.612 vs 0.619). Collectively, ROC curve analyses suggested that SIRI possessed acceptable sensitivity and specificity for prognostic evaluation of 30-day all-cause mortality among AECOPD patients.

**Figure 3.**
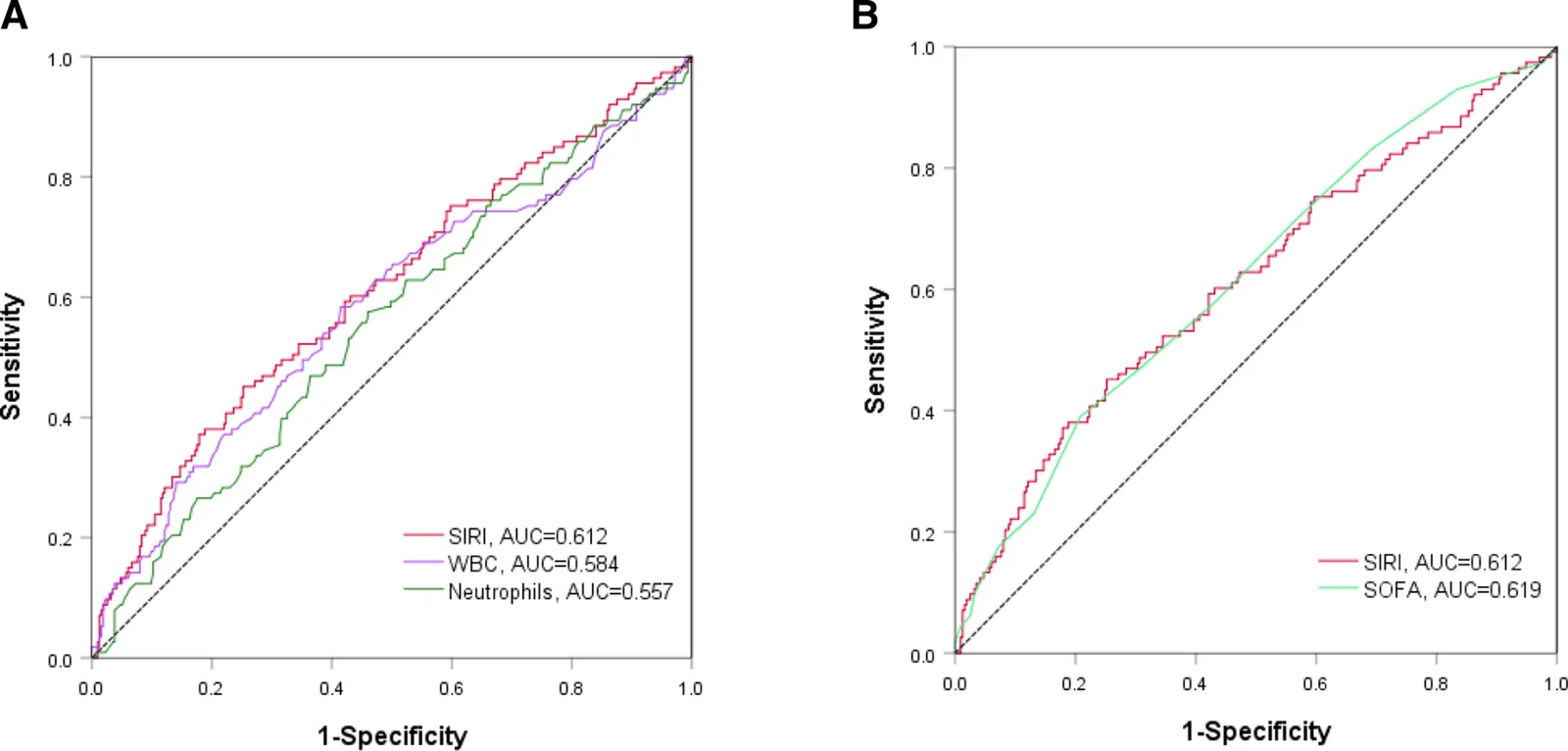
The receiver operating characteristic curves of predictive value of different indicators for 30-day mortality in patients with AECOPD. AECOPD = acute exacerbation of chronic obstructive pulmonary disease, AUC = area under the curve, SIRI = systemic inflammation response index, WBC = white blood cell, SOFA = sequential organ failure assessment.

## 4. Discussion

This retrospective study mainly explored the prognostic relationship between SIRI and clinical outcomes in AECOPD patients, including 30-day, 90-day, and 1-year all-cause mortality and in-hospital mortality. After adjustment for multiple confounding variables, Cox regression analyses confirmed that elevated SIRI constituted an independent risk factor for 30-day all-cause mortality in AECOPD patients. Subgroup analyses across diverse clinical covariates and comorbidities revealed no significant interaction terms, further supporting the reliability of our conclusions.

As described above, COPD is associated with high global mortality, and AECOPD episodes further increase the risk of death. Hence, identifying prognostic factors for AECOPD represents a challenging yet clinically meaningful task for pulmonologists. Numerous studies have reported prognostic factors for AECOPD, including the severity of COPD at baseline, frequency of exacerbations, age, various comorbidities, prior hospitalization history, and a range of physiological and laboratory parameters such as lung function, respiratory rate at admission, hypoxemia, and hypercapnia.^[25,26]^Nevertheless, clinicians still pursue more practical mortality-predictive biomarkers, especially markers readily available upon hospitalization for AECOPD patients. Oren Fruchter et al.^[27]^ conducted a retrospective analysis of 61 AECOPD patients, concluding that elevated D-dimer is a reliable prognostic indicator for both short-term and long-term survival in AECOPD patients.Additionally, a study by Zemans et al^[28]^ indicated that composite clinical indicators may predict COPD prognosis better than individual clinical indicators, which has increased interest in composite clinical metrics. Zixiong Zeng et al^[29]^ conducted multivariate logistic regression analysis and multivariate Cox regression analysis on 362 patients, concluding that an elevated blood urea nitrogen to albumin ratio is a strong and independent predictor of in-hospital mortality and 90-day all-cause mortality in patients with AECOPD. Moreover, the study by Shuai Shao et al.^[30]^ showed that the neutrophil-to-lymphocyte ratio could serve as a biomarker for predicting in-hospital mortality, long-term mortality, treatment failure, organ failure, and readmission within 15 months for AECOPD patients.Whether using single clinical indicators or composite clinical indicators, clinicians aim to achieve the most accurate predictive results using the simplest methods.

Previous research indicates that the characteristics of COPD include both local and systemic abnormalities and persistent inflammation. This inflammatory response involves both innate and adaptive immune reactions.^[31]^ As a composite metric integrating 3 inflammatory cell subsets, SIRI enables comprehensive assessment of innate and adaptive immune status. Since SIRI is derived from routine complete blood count tests, it can be conveniently and cost-effectively obtained for risk evaluation among hospitalized patients. Furthermore, some researchers believe that SIRI, by combining neutrophils, monocytes, and lymphocytes, can better reflect the body’s inflammatory and immune status. The ratio can represent the balance between inflammatory activation factors and regulatory factors: a higher SIRI ratio indicates greater imbalance, suggesting a more severe inflammatory response and stronger immune suppression.^[32]^ This aligns with the findings of this study, which show that among patients with AECOPD, the mortality rate is higher in the high SIRI group compared to the low SIRI group. Importantly, the further results of this study indicate that SIRI is an independent risk factor for 30-day all-cause mortality in AECOPD patients and has good predictive value for this outcome.

Several limitations of this study need to be acknowledged. First, this is a single-center retrospective observational study with a moderate sample size. Second, only laboratory parameters collected within 24 hours after ICU admission were analyzed; we did not investigate dynamic temporal changes of SIRI during hospitalization and their prognostic implications for AECOPD-related mortality. Third, we failed to account for potential influences of specific therapeutic regimens such as corticosteroid administration on patient prognosis. Therefore, our findings require further validation by large-scale multicenter prospective studies in the future.

## 5. Conclusion

The present study suggests that SIRI serves as a promising novel inflammatory biomarker for predicting 30-day all-cause mortality risk in patients with AECOPD. Elevated SIRI correlates with increased all-cause mortality risk and represents an independent risk factor for 30-day all-cause mortality among AECOPD patients. Risk stratification based on SIRI may facilitate early identification of high-risk AECOPD patients and guide timely targeted clinical interventions to improve patient prognosis and quality of life.

## Author contributions

**Conceptualization:** Yahu Bai.

**Data curation:** Yahu Bai.

**Writing – original draft:** Yahu Bai.

**Writing – review & editing:** Yahu Bai.
